# Mindfulness-induced endogenous theta stimulation occasions self-transcendence and inhibits addictive behavior

**DOI:** 10.1126/sciadv.abo4455

**Published:** 2022-10-12

**Authors:** Eric L. Garland, Adam W. Hanley, Justin Hudak, Yoshio Nakamura, Brett Froeliger

**Affiliations:** ^1^ Center on Mindfulness and Integrative Health Intervention Development, College of Social Work, University of Utah, Salt Lake City, UT, USA.; ^2^ Veterans Health Care Administration VISN 19 Whole Health Flagship site located at the VA Salt Lake City Health Care System, Salt Lake City, UT, USA.; ^3^ Department of Anesthesiology, University of Utah School of Medicine, Salt Lake City, UT, USA.; ^4^ Department of Psychology, University of Missouri, Columbia, MO, USA.

## Abstract

Self-regulation is instantiated by theta oscillations (4 to 8 Hz) in neurons of frontal midline brain regions. Frontal midline theta (FMΘ) is inversely associated with default mode network (DMN) activation, which subserves self-referential processing. Addiction involves impaired self-regulation and DMN dysfunction. Mindfulness is an efficacious self-regulatory practice for treating addiction, but little is known about the mechanisms by which mindfulness reduces addictive behavior. In this mechanistic study of long-term opioid users (*N* = 165), we assessed meditation-induced FMΘ as a mediator of changes in opioid misuse. Relative to a supportive psychotherapy control, participants treated with Mindfulness-Oriented Recovery Enhancement (MORE) exhibited increased FMΘ during a laboratory-based meditation session. FMΘ during meditation was associated with self-transcendent experiences characterized by ego dissolution, nondual awareness, and bliss. MORE’s effects on decreasing opioid misuse were mediated by increased FMΘ. Given the role of aberrant self-referential processing in addiction, mindfulness-induced endogenous theta stimulation might “reset” DMN dysfunction to inhibit addictive behavior.

## Teaser

Mindfulness meditation reduced addictive behavior by increasing theta oscillations in the brain.

## INTRODUCTION

Self-regulation of goal-oriented behavior results from adaptive cognitive control functions in the human brain, mediated by widespread neural communications centered on the hub of the prefrontal cortex (PFC) (*1*, *2*). In particular, frontal midline brain regions [e.g., medial PFC (mPFC) and anterior cingulate cortex (ACC)] appear essential to adaptive control, especially during modulation of reward value (*3*, *4*) and regulation of self-relevant distractors (*5*)—processes integral to action selection and goal attainment. Frontal midline theta (FMΘ) oscillations (4 to 8 Hz) are an established mechanism of adaptive control (*6*) instantiated by medial prefrontal brain regions (*7*). FMΘ is inversely associated with activation in the default mode network (DMN) (*8*), known to subserve self-referential processing during resting states (*9*). Thus, FMΘ increases when self-referential processing is suspended during deep cognitive absorption with a non–self-referential task state (*10*). Moreover, inducing FMΘ through exogenous brain stimulation has been shown to enhance adaptive control over behavior (*11*). Putatively, FMΘ stimulation may therefore enhance adaptive control of aberrant behavior in conditions known to involve DMN dysfunction, reward dysregulation, and maladaptive self-referential processing, including depression (*12*), chronic pain (*13*), and addiction (*14*). In addiction, adaptive cognitive control is usurped by drug-related reward processing, whereby drug cues are overvalued, and thereby elicit prepotent drug-seeking responses at the expense of homeostatic goal pursuit via salutogenic natural rewards (*15*).

Mindfulness-based interventions (MBIs) may treat addictive behaviors by providing a means of endogenous FMΘ stimulation. MBIs include training in focused attention and open monitoring meditation techniques that recruit multiple adaptive control processes (*16*). Focused attention meditation involves sustained attention on the target of mindfulness (usually breath and body sensations), executive attention to notice and reduce mind wandering into self-referential thought, and attentional reorienting back to the target. Building on these adaptive control processes, open monitoring meditation involves a form of ambient attention and meta-awareness that monitors the flux of self-referential mental content while reflecting upon the “background” of awareness in which this content arises. As open monitoring reaches its apex, mindfulness practitioners sometimes report profound changes in self-referential processing, characterized by ego dissolution and an experiential nondual awareness of the lack of distinction between subject and object (*17*), states that are often marked by an affective qualia of bliss (*18*). Although such meditation-induced self-transcendent states are usually assumed to be achieved by only expert practitioners with tens of thousands of hours of meditation experience (*19*, *20*), psychometric research indicates that they can occur even among novices (*21*, *22*).

Electroencephalography (EEG) has identified FMΘ as a primary biomarker of mindfulness meditation (*23*, *24*) and the self-transcendent state produced by such meditative practice (*25*). FMΘ power is thought to mediate recruitment of meta-awareness and cognitive control to sustain the meditative state (*26*). FMΘ coherence, wherein simultaneously measured EEG channels synchronize their activity at a theta frequency, has been observed in multiple studies of mindfulness meditation (*24*, *27*–*31*). FMΘ power and coherence are believed to index mPFC and ACC activation during the deepening of the meditative state and to be linked with increased functional and structural plasticity in these brain regions (*27*, *31*). Hypothetically, meditation-induced self-transcendent states of sufficient depth to suspend self-referential processing may produce endogenous theta stimulation of the PFC, thereby mediating the effect of MBIs on adaptive control over dysfunctional behavior, such as substance misuse (*32*).

Here, we aimed to test this hypothesis in a mechanistic study examining Mindfulness-Oriented Recovery Enhancement (MORE) (*33*) versus a Supportive Group (SG) Psychotherapy control condition. MORE is an evidence-based treatment that integrates mindfulness meditation with cognitive and affective techniques designed to ameliorate addiction, emotional distress, and physical pain (*34*). Building upon our preliminary work where we first demonstrated that mindfulness increased FMΘ in a pilot sample of chronic opioid users (*25*), in the present investigation, we sought to test mindfulness-induced changes in FMΘ as a mediator of the effects of MORE on long-term reductions in addictive behavior in a substantially larger sample of opioid misusing patients (*N* = 165). Opioid misuse, consisting of aberrant drug-related behaviors inconsistent with prescription directions (e.g., opioid dose escalation or self-medication of negative affect with opioids), affected more than 9 million Americans in 2020 (*35*) and has helped fuel the ongoing opioid crisis in the United States (*36*). MORE was designed as a targeted intervention for opioid misuse, and large-scale clinical trials have found MORE to be highly efficacious, more than doubling the effect of standard supportive psychotherapy on reducing opioid misuse (*34*). For the present investigation, we hypothesized that patients treated with MORE would exhibit increased FMΘ spectral power and coherence during a laboratory-based mindfulness meditation session that would be associated with increases in self-transcendence and statistically mediate the effect of MORE on decreases in opioid misuse.

## RESULTS

**Fig. 1.**
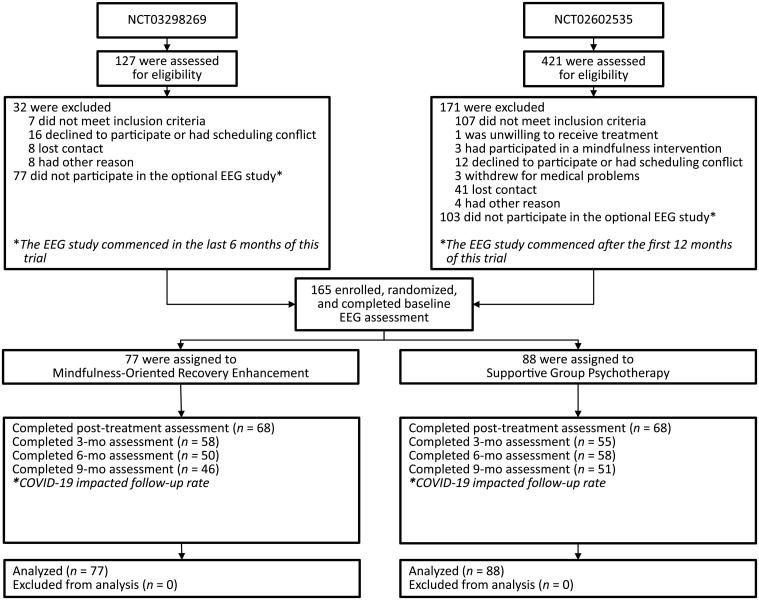
CONSORT diagram depicting participant flow through the study. This mechanistic EEG study involved patients recruited from two preregistered clinical trials (ClinicalTrials.gov identifiers NCT02602535 and NCT03298269). Because the EEG protocol was added later after enrollment for these trials had commenced, a subset of participants enrolled in the primary clinical trials participated in this mechanistic study.

We obtained data from 165 patients with long-term opioid use (65.2% female, mean age = 54.3, SD = 11.1 years) and chronic pain (mean pain severity = 6.1, SD = 1.9). The mean duration of opioid use in the sample was 114.7 months (range, 4 to 480). At baseline, the mean (±SE) opioid dose of the sample in morphine milligram equivalents (MME; 100.0 mg, SD = 152.4 mg) fell within the “high-dose” range (>90 MME) as specified by the Centers for Disease Control (*37*). At baseline, the mean opioid misuse score on the Current Opioid Misuse Measure (COMM) was 16.7 (SD = 7.7). COMM scores ≥ 9 indicate the presence of opioid misuse (*38*). See Table 1 for other demographic and clinical characteristics and Fig. 1 for the CONSORT study flow diagram.

**Table 1.** Demographic and clinical characteristics of the sample of opioid-treated chronic pain patients (*N* = 165).

**Measure**	**Mindfulness-Oriented Recovery Enhancement (*n* = 77)**	**Supportive Group Psychotherapy (*n* = 88)**
Age	54.7 ± 11.7	53.9 ± 10.5
Female, *N* (%)	48 (64%)	58 (66%)
Pain condition/ location, *N* (%)*		
Back pain	43 (57%)	51 (58%)
Osteoarthritis pain	7 (9%)	12 (14%)
Fibromyalgia	7 (9%)	4 (5%)
Neuropathic pain	6 (8%)	5 (6%)
Cervical pain	1 (1%)	3 (3%)
Extremity pain	2 (3%)	2 (2%)
Other	5 (6%)	7 (8%)
Pain severity (0–10)	6.3 ± 1.7	5.9 ± 2.1
Pain duration in years	14.6 ± 10.4	15.1 ± 10.1
Primary opioid type, *N* (%)		
Hydrocodone	18 (23%)	20 (23%)
Oxycodone	25 (32%)	29 (33%)
Tramadol	12 (16%)	14 (16%)
Morphine	10 (13%)	7 (8%)
Fentanyl	1 (1%)	2 (2%)
Methadone	2 (3%)	7 (8%)
Buprenorphine	2 (3%)	3 (3%)
Other	1 (1%)	0 (0%)
Duration of opioid use in months	109.4 ± 89.0	119.4 ± 101.0
Morphine equivalent daily dose in milligrams	81.6 ± 121.4	115.9 ± 174.0
Opioid misuse, COMM score	16.1 ± 7.5	17.2 ± 7.9
Opioid use disorder diagnosis	40 (53%)	60 (71%)
Positive drug urine screen^†^	14 (26%)	11 (20%)

*Because patients could report multiple pain conditions and locations, this percentage could sum to greater than 100%.

†A positive drug urine screen was given if a participant tested positive for illicit drugs or for a nonprescribed opioid medication.

**Fig. 2.**
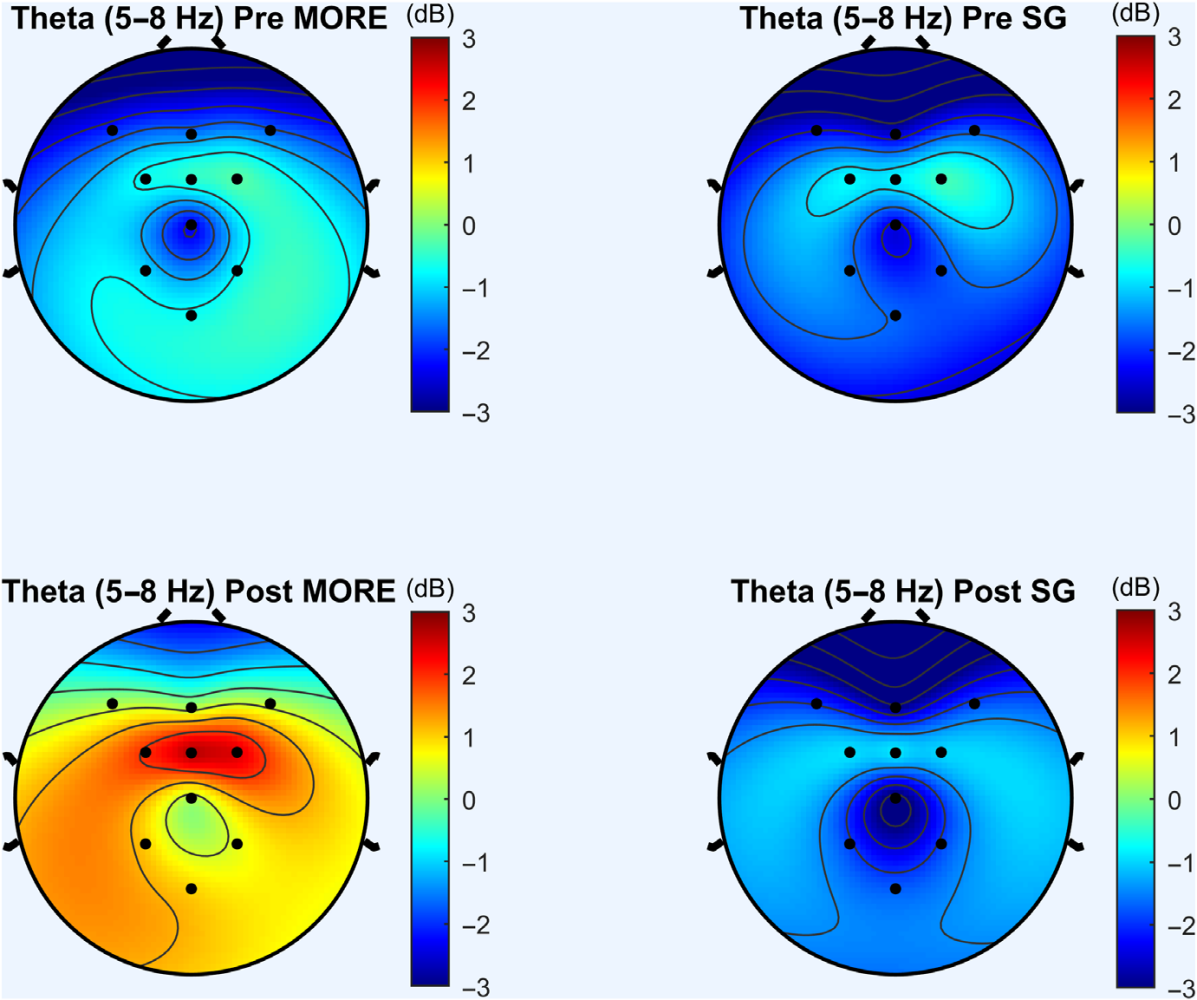
Theta power topomaps for patients in Mindfulness-Oriented Recovery Enhancement (MORE) and Supportive Group (SG) Psychotherapy at pre- and posttreatment. Increases in FMΘ EEG power were observed in the MORE group at posttreatment, relative to the SG Psychotherapy control group.

**Fig. 3.**
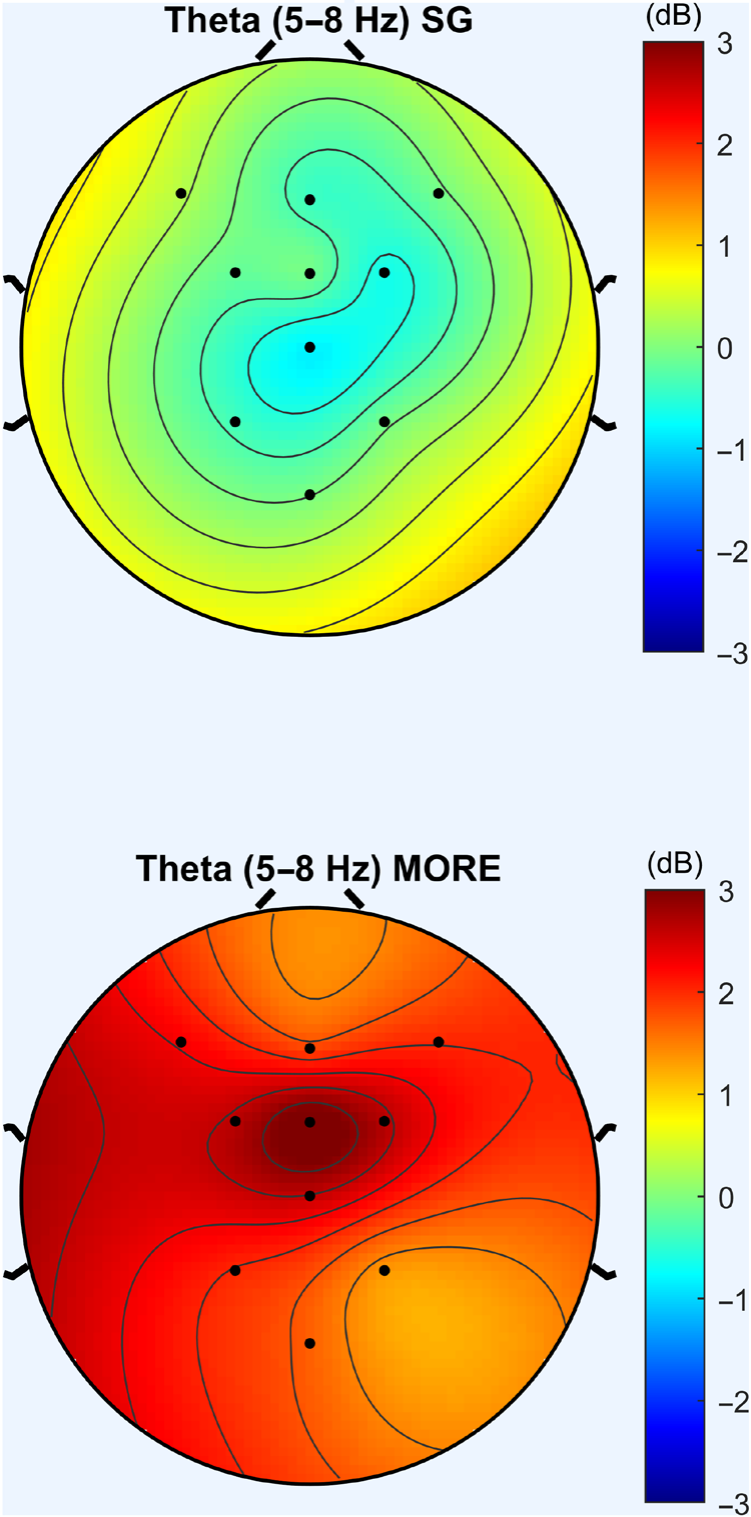
Mindfulness-Oriented Recovery Enhancement (MORE) produced significantly greater increases in meditation-induced FMΘ EEG power by posttreatment than the Supportive Group (SG) Psychotherapy control condition. Power topomaps represent change from pre- to posttreatment during the laboratory-based meditation session.

**Fig. 4.**
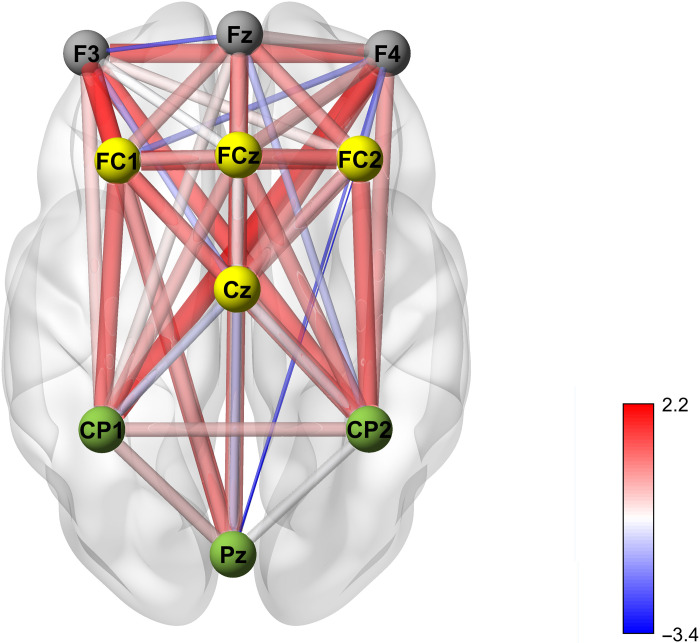
Mindfulness-Oriented Recovery Enhancement (MORE) produced significantly greater increases in meditation-induced FMΘ EEG coherence by posttreatment than the Supportive Group (SG). Psychotherapy control condition. Coherence map represents the MORE versus SG between-group effect on change in theta coherence during the laboratory-based meditation session from pre- to posttreatment.

Relative to the SG, MORE was associated with significantly greater increases in FMΘ spectral power (*F*_1,110.929_ = 10.96, *P* = 0.001; Figs. 2 and 3) and coherence (*F*_1,127.82_ = 6.56, *P* = 0.012; Fig. 4) during the laboratory-based mindfulness meditation practice session. In a sensitivity analysis controlling for baseline opioid use disorder diagnosis and opioid dose, participants in MORE continued to exhibit significantly greater increases in FMΘ power (*F*_1,104.67_ = 9.64, *P* = 0.002) and coherence (*F*_1,119.46_ = 6.71, *P* = 0.011) than the SG.

**Fig. 5.**
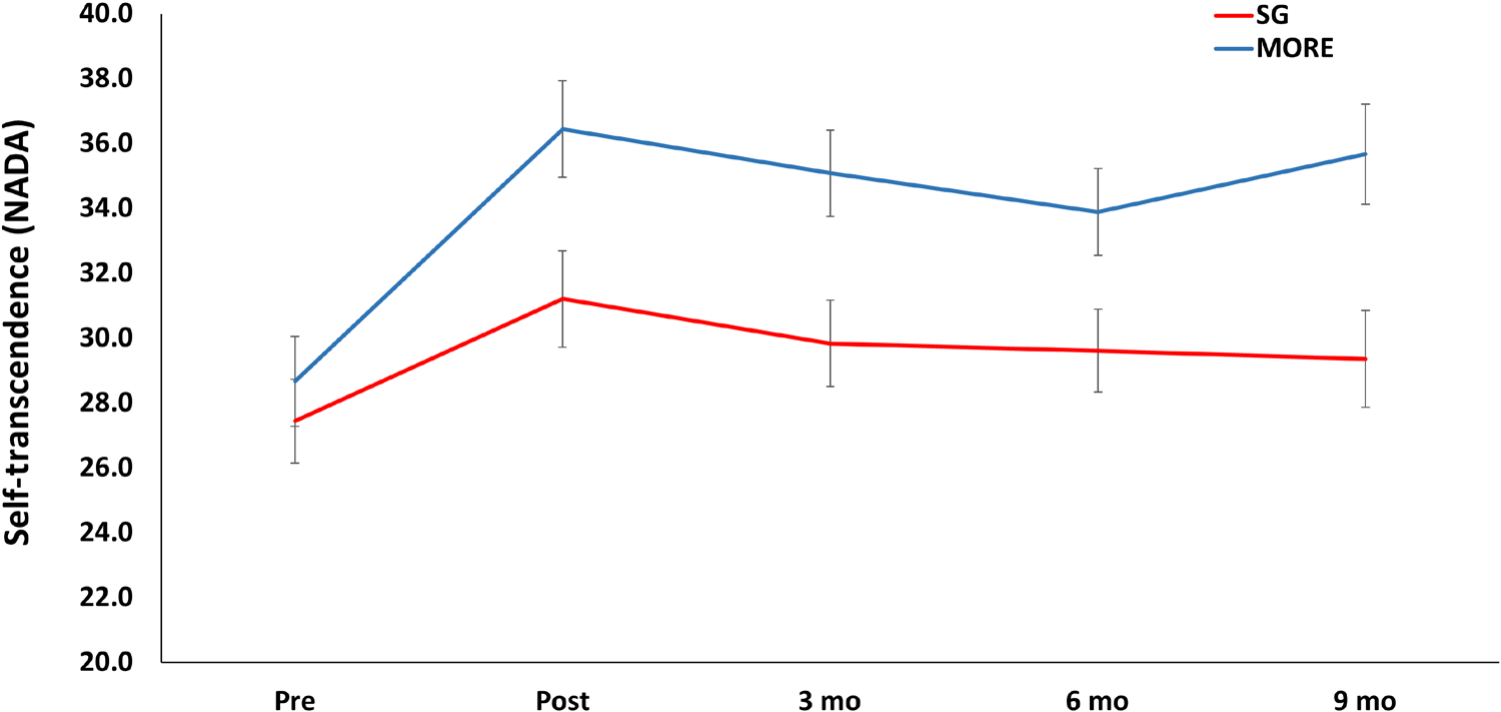
Mindfulness-Oriented Recovery Enhancement (MORE) produced significantly greater increases in self-transcendence than the Supportive Group (SG) Psychotherapy control condition. Self-transcendence was measured with the Nondual Awareness Dimensional Assessment (NADA) through 9-month follow-up.

MORE also produced significantly greater increases in self-transcendent experiences [indexed by the Nondual Awareness Dimensional Assessment–Trait (NADA) (*21*)] than the SG through 9-month follow-up (*F*_1,125.87_ = 6.43, *P* = 0.012; Fig. 5). That is, participants in MORE reported an enhanced propensity to experience ego dissolution and a phenomenological unity between self and world coupled with affective bliss; this propensity was sustained 9 months after the end of the MORE intervention. Increased FMΘ power during the laboratory-based mindfulness mediation practice significantly predicted increases in self-transcendent experiences (*B* = 0.39, SE = 0.16, *P* = 0.01).

**Fig. 6.**
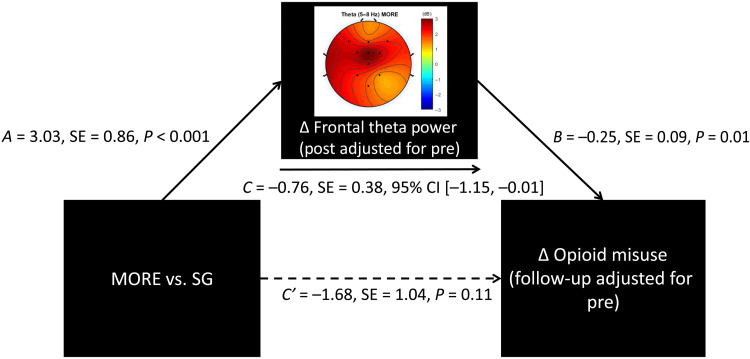
Path model testing FMΘ power as a mediator of reduced opioid misuse. Path model indicating that the effect of Mindfulness-Oriented Recovery Enhancement (MORE) versus Supportive Group (SG) Psychotherapy control condition on reducing opioid misuse through 9-month follow-up was statistically mediated by increasing FMΘ power during mindfulness meditation.

MORE also produced significantly greater reductions in opioid misuse [indexed by the COMM (*38*)] than the SG through 9-month follow-up (*F*_1,145.62_ = 4.87, *P* = 0.029; Table 2). By 9-month follow-up, the mean opioid misuse score in participants treated with MORE decreased to levels beneath those pathognomonic for opioid use disorder (*39*), whereas participants in the SG continued to exhibit opioid misuse scores at levels consistent with opioid use disorder. The effect of MORE on opioid misuse in the follow-up period was statistically mediated by increases in FMΘ power [*B* = −0.76; SE = 0.38; 95% confidence interval (CI) = −1.51 to −0.01; Fig. 6] and FMΘ coherence (*B* = −0.61; SE = 0.29; 95% CI = −1.12 to −0.02), as indicated by the 95% CI of the indirect effect estimate not spanning zero in both mediation models.

**Table 2.** Effects of Mindfulness-Oriented Recovery Enhancement (MORE) versus Supportive Group (SG) Psychotherapy on the COMM. Data are reported as estimated marginal mean (SE).

	**Pre**	**Post**	**3 months**	**6 months**	**9 months**
MORE	16.12 (1.01)	11.74 (0.87)	11.28 (0.91)	10.44 (1.01)	11.22 (1.08)
SG	17.20 (0.94)	15.34 (0.85)	14.30 (0.90)	12.81 (0.95)	13.29 (1.03)

## DISCUSSION

Mindfulness meditation is a self-regulatory practice involving the training of meta-awareness for monitoring and adaptive control of attention and self-referential thought (*27*, *40*). MBIs have emerged as promising treatments for addiction, with meta-analytic evidence demonstrating their efficacy across a range of addictive behaviors (*32*). Here, we showed using a randomized controlled design with an active control group that training in mindfulness meditation through the MORE intervention generated robust endogenous theta stimulation in frontal midline brain regions. In turn, mindfulness-induced theta oscillations were associated with self-transcendence and mediated the effect of MORE on reduced opioid misuse—a pernicious and prevalent public health concern being addressed with heightened urgency at both clinical and policy levels.

Prior research demonstrates that exogenous stimulation of theta activity in the PFC enhances cognitive control capacity to promote adaptive behavior (*11*). FMΘ predicts the propensity for enhanced adaptive control over prepotent actions (*41*), whereas impaired control over prepotent addictive responses elicited by conditioned interoceptive (e.g., emotional distress) and exteroceptive stimuli (e.g., the sight of an opioid pill bottle) contributes to addiction vulnerability (*15*). FMΘ is instantiated by medial prefrontal brain regions including mPFC and ACC (*7*), and MBIs have been shown to boost neural activity and efficiency of white matter connectivity in these brain structures (*31*). Hypothetically, mindfulness-induced FMΘ might mediate adaptive cognitive control via stimulation of midline brain regions and modulation of neural plasticity and functional connectivity (*31*). In the present study, mindfulness meditation amplified FMΘ power and coherence—neural effects that were associated with reduced opioid misuse. Thus, mindfulness meditation, as a form of mental training, may have strengthened adaptive cognitive control capacity by endogenously stimulating theta oscillations in the PFC, thereby helping patients to exert self-control over their addictive behavior. Paralleling our results, exogenous theta stimulation appears to facilitate inhibitory control of substance use and misuse. For example, among smokers, excitatory theta burst stimulation (TBS) to the PFC significantly increases inhibitory control (*42*)—a neurocognitive mechanism that predicts decreased smoking relapse vulnerability (*43*). In patients with methamphetamine use disorder, TBS over the PFC has also been shown to improve cognitive function and reduce craving (*44*), as well as to reduce functional connectivity with the DMN (*45*). Thus, integrative therapies combining exogenous (i.e., TBS and/or neurofeedback technologies) and endogenous means of theta stimulation (e.g., via mindfulness meditation) might produce an especially efficacious treatment for opioid misuse, opioid use disorder, and other substance use disorders.

FMΘ also increases during suppression of DMN activity (*8*). In that regard, mindfulness meditation is thought to involve intensive recruitment of cognitive control resources (*27*) to inhibit self-referential thought during the focus of attention on the meditative object (e.g., the breath). The repeated cultivation of one-pointed attention during meditation is thought to deepen into a state of absorption (*samādhi* in Sanskrit). When attentional absorption reaches its zenith and self-referential thought is quelled, attention can be finally released as the subject-object distinction falls away during what is purported to be a transcendent experience of the background of awareness (*17*). As articulated in the 10th century Tantric text *The SpandaVivrti*, “At the time when the adept pervades all things that exist on the plane of objectivity by his cognitive intent, and envelops them with the light of his own consciousness so as to internalize them within it, his erroneous perception of multiplicity ceases, and so he brings all things to rest together in the one reality” (*46*). Here, we found that 8 weeks of an MBI, MORE, significantly increased the propensity to experience self-transcendence, effects that were maintained 9 months after the end of the intervention.

Furthermore, increases in FMΘ power during mindfulness meditation occasioned more profound self-transcendent experiences. Our results are congruent with findings from earlier studies, indicating that higher levels of FMΘ power during meditation are associated with increasingly deep meditative states characterized by bliss, absorption, a oneness between subject and object, and dissolution of the sense of self (*10*, *25*, *28*, *29*). The phenomenology of transcendence—marked by a sense of nonduality between subject and object and a rapturous affective tone of blissful reward—might not be mere epiphenomena but rather track with the mechanistic changes occasioned by disciplined mindfulness practice. That is, insofar as transcendence attenuates default mode self-referential processing (*47*), the filter that typically parses stimulus salience for self-relevance may be disinhibited, resulting in natural reward and apperception of affective meaning across the sensory-perceptual field (*48*). Self-transcendence and nondual awareness might be reflected in increased functional connectivity between dorsal attentional, default mode, and salience networks observed during the practice of meditation (*49*). According to 10th century Kashmiri and Tibetan meditation treatises (*46*, *50*), when freed from self-referential processing, awareness can turn back upon itself in a reflexive act to appreciate its own inherent salience and, in so doing, abolish any sense of craving, desire, or lack. As expressed in the *Sivasutra*, “When the yogi is established in pure awareness, his craving is destroyed… thus he savors his own inherently blissful nature which illumines itself with the rays of its consciousness… Thus [at] the very moment the yogi abandons the craving.” (*51*). Because DMN activity is inversely associated with dopamine signaling in the brain (*52*), hypothetically, meditation-induced disruption of attractor networks in the DMN may disinhibit endogenous dopaminergic activity, resulting in increased reward prediction error and novelty detection that produce intrinsic reward states potent enough to outweigh the pull of drug-related reward (*53*, *54*). Given the role of aberrant self-referential processing in addictive behavior (*14*), endogenous theta stimulation produced during transcendent meditative states might “reset” the DMN dysfunction that undergirds addiction. That said, nondual meditation in long-term practitioners has also been associated with increased gamma power (*20*); it is plausible that accessing the nondual state in novices required substantial cognitive effort, as manifested by FMΘ, whereas adepts can achieve this state in an effortless manner, as indexed by heightened gamma synchrony.

We observed a significant reduction in opioid misuse following treatment with MORE relative to the supportive psychotherapy control condition. It should be noted that in the current study, opioid misuse was measured by a validated self-report instrument (COMM). We selected this assessment approach for our analysis because it yields a continuous measure of opioid misuse, thereby providing greater statistical power to detect its associations with FMΘ. However, in the parent clinical trial (*N* = 250) associated with the present mechanistic study, MORE reduced occurrence of opioid misuse, as objectively measured by a composite binary index triangulating self-reports on the COMM, blinded clinical interview, and drug urine screen, by 45% at the 9-month follow-up (*34*). The present study results replicate findings from several other trials showing that MORE reduces opioid use and misuse (*25*, *55*–*58*). Together, these data demonstrate MORE’s sustained efficacy as a treatment for opioid misuse.

Last, a high-density electrode montage for source localization would have permitted strong inferences about the brain networks stimulated by mindfulness meditation. However, we selected a limited set of electrodes to minimize participant burden, in light of the vulnerable nature of the study participants. Furthermore, although we observed increased meditation-induced FMΘ coherence following treatment with MORE, these coherence findings may be secondary to FMΘ power increases rather than a reflection of heightened neural synchrony in frontal midline brain structures. Functional neuroimaging (e.g., functional magnetic resonance imaging) is now indicated to resolve these outstanding mechanistic research questions. Regardless of its underlying neural generators, we speculate that FMΘ may serve as a highly accessible biomarker of meditative state depth and signal treatment response for patients with addictive behaviors.

## MATERIALS AND METHODS

This was an ancillary mechanistic study of patients recruited from two preregistered clinical trials (ClinicalTrials.gov identifiers NCT02602535 and NCT03298269). Participants (*N* = 165) met study inclusion criteria if they (i) had chronic pain and (ii) had taken opioid analgesics daily or nearly every day for at least the past 90 days. Participants were invited to complete a laboratory-based mindfulness meditation session 1 week before and 1 week after participating in an 8-week MORE or SG intervention. For the ancillary mechanistic study reported here, we added an EEG protocol to these two already existing clinical trials where EEG outcomes were not proposed as part of the original clinical trial designs. Subsequent to the funding of the grants that supported these trials, we later had the opportunity to add the EEG protocol described here as an ancillary mechanistic study overlaid on the clinical trial. As such, only a subset of participants enrolled in the primary clinical trials had the opportunity to participate in this mechanistic study. Although opioid misuse was the primary outcome of the clinical trials from which the present study sample was recruited, the primary outcome of this mechanistic study was FMΘ activity. Following an intent-to-treat approach, we included participants in the present mechanistic analysis if they began the EEG study, regardless of treatment completion. Following informed consent, and again at posttreatment, participants completed the meditation session while EEG was recorded, as well as a validated 17-item measure of opioid misuse, the COMM (*26*). The COMM, which assesses a range of aberrant drug-related behaviors associated with opioid misuse (e.g., unauthorized dose escalation and use of opioids to self-medicate negative affect), has been widely used to assess changes in opioid misuse and exhibits strong convergent validity with clinical interview and urine drug screen evidence of opioid misuse (*59*). Participants also completed a validated 13-item self-report instrument measuring experiences of self-transcendence, the NADA (*21*), which assesses experiences of ego dissolution, unity, and corresponding blissful affective states. After the pretreatment assessment, participants were randomly allocated to MORE or the SG. The allocation sequence was generated via computerized random number table by a researcher who was uninvolved in assessment, treatment, or enrollment using simple randomization in blocks of varying sizes to preserve allocation unpredictability. Assessments were conducted by research staff blinded to group assignment (which remained concealed throughout the study). The COMM and NADA were also completed at 3-, 6-, and 9-month follow-ups. The protocol was approved by the university institutional review board; all procedures complied with the Helsinki Declaration. Participants were excluded if actively suicidal or psychotic. There were no significant between-group differences in demographic or baseline clinical characteristics save for opioid use disorder diagnosis, which by chance was higher in the SG. Participants were compensated for completing the study.

### Mindfulness-Oriented Recovery Enhancement

MORE was delivered as a manualized eight-session group intervention (*33*) designed to address mechanisms implicated in opioid misuse. MORE sessions provided mindfulness training to promote self-awareness and self-regulation of automatic addictive habits. Mindfulness training involved mindful breathing and body scan techniques, with instructions to deepen meta-awareness into nondual states of consciousness imbued with qualities of self-transcendence—i.e., a dissolving of the sense of self and/or a oneness between self and world with attendant positive affective qualities of peace, awe, or bliss (*21*). MORE also provided training in reappraisal to regulate negative emotions and promote meaning in the face of adversity, and training in savoring to amplify natural reward processing and boost positive emotions. MORE session recordings were monitored for treatment fidelity via the MORE Fidelity Measure (*55*); a consistently high level of fidelity was attained. Participants were asked to engage in daily 15-min mindfulness sessions at home guided by an audio track. In addition, participants were asked to engage in mindful breathing before making a decision about whether or not to use opioids. This exercise was intended to clarify whether opioid use was driven by craving versus a need for pain relief, and to prevent unnecessary opioid dosing by providing a nonpharmacologic pain management approach.

### Supportive Group

The time-matched active control condition in this study consisted of a manualized, eight-session SG treatment, in which a clinician facilitated emotional expression and discussion of topics pertinent to chronic pain and opioid use/misuse. Session recordings were reviewed to monitor adherence to the SG treatment manual; a consistently high level of treatment fidelity was achieved. To match the MORE homework requirement, SG participants were asked to engage in 15 min of journaling a day on chronic pain-related themes.

### EEG during the laboratory-based mindfulness meditation session

EEG was continuously recorded from 10 midline scalp sites (Fz, F3, F4, FC1, FC2, FCz, Cz, CP1, CP2, and PZ) using an active sensor cap with Ag/AgCl electrodes (actiCap GmbH, Herrsching, Germany). In addition, vertical electrooculograms were recorded. All recordings were collected with an actiCHamp amplifier (Brain Products GmbH, Gilching, Germany). Data were acquired at a sampling rate of 500 Hz, a resolution of 0.489 μV, and an amplification cutoff of 140 Hz, with impedances kept below 10 kilohms. EEG was recorded during a 10-min mindfulness meditation session. All participants received the same session instruction: “Now practice mindfulness, which means focusing on your thoughts, feelings and body sensations in the present moment in a nonjudgmental way, without reacting to them.” In keeping with methods used in previous mindfulness studies (*25*, *57*), to control for demand characteristics, these mindfulness instructions were kept constant across both treatment conditions (MORE and SG), allowing us to isolate the effects of mindfulness training through the MORE intervention from any potential instruction effects. We assumed that meditation-naïve participants randomized to the SG control would be unable to successfully practice mindfulness meditation with such nondescript instructions, whereas participants randomized to MORE would be cued by these simple instructions to practice the mindfulness technique taught during the 8-week MORE intervention. The meditation session was composed of two 5-min blocks (eyes open and eyes closed). We computed average EEG spectral frequency power and coherence values across the 5-min eyes closed portion of the meditation at the pre-assessment. We then computed average EEG power and coherence values across the 5-min eyes closed portion of the meditation at post-assessment.

### Data reduction

All EEG analyses were performed in MATLAB using scripts implementing the EEGLAB toolbox (*60*). In a first step, epochs of 300 s were created for each subject. Then, the signal was notch-filtered at 60 Hz, low pass–filtered at 40 Hz cutoff using a fourth-order Butterworth filter, and high pass–filtered at 0.1 Hz cutoff using a second-order Butterworth filter. Filtered data were passed through the PREP pipeline (*61*), which detrends the data, applies a notch filter tapering off the harmonics of 60 Hz, re-references the data to the linked earlobe, and identifies and interpolates bad channels (<10% of data were interpolated). Interpolation in low-density montages has been shown to produce valid data (*25*, *61*, *62*). In the power spectral density analyses, average power spectra in the theta (4 to 8 Hz) band were calculated using Welch’s periodogram. Coherence values were calculated using the mscohere function in MATLAB. Briefly, this function computes the magnitude of squared coherence, a function of the power spectral densities, and the cross-power spectral density of two signals, *x* and *y*, calculated using Welch’s overlapped averaged periodogram method. Theta coherence values were averaged over the relevant spectra (4 to 8 Hz). These averages, both power and coherence, were then averaged again over regions of interest (ROIs) specified in prior mindfulness studies (*28*, *29*): frontal (F3, F4, and Fz), frontal midline (FMΘ: FCz, FC1, FC2, and Cz), and parietal (Pz, CP1, and CP2). Per our a priori hypothesis, our analysis focused on the frontal midline ROI. Separate sensitivity analyses of the EEG data were conducted after removing artifacts identified by visual inspection, and this did not alter the significance or directionality of the hypothesized treatment effects.

### Statistical analyses

We undertook a stepwise analytic approach. To assess effects of treatment on FMΘ power and coherence, we used mixed modeling with maximum likelihood estimation and fixed effects consisting of a time factor and between-subject treatment factor (MORE versus SG). Next, we used a similar mixed modeling approach to assess effects of treatment on self-transcendence and opioid misuse outcomes, adjusting for prerandomization baseline values. Here, the primary fixed effect of interest was the adjusted treatment main effect, which estimates the overall benefit of MORE versus SG averaged across all follow-ups. Because patients in the SG had higher mean COMM scores at prerandomization baseline, baseline adjustment was warranted. Then, we used a mixed model to test the fixed effect of FMΘ power increases during meditation as a predictor of changes in self-transcendence. Mixed models were specified with random intercepts, a diagonal covariance structure, and Satterthwaite adjusted degrees of freedom. Last, we conducted a path analysis in R-Lavaan to test whether the effects of MORE on opioid misuse (the overall benefit of MORE versus SG averaged across all follow-ups, in accordance with our mixed model above) were mediated by pre-post increases in FMΘ power and coherence, with significant mediation indicated by the 95% bias-corrected CIs not spanning zero.

## Notes

View/request a protocol for this paper from *Bio-protocol*.
