# Supplementary material for: Mindfulness-induced endogenous theta stimulation occasions self-transcendence and inhibits addictive behavior
Source: Sci Adv. 2022 Oct 12;8(41):eabo4455. doi: 10.1126/sciadv.abo4455 (PMC9555770; doi:10.1126/sciadv.abo4455)

Science Advances
AAAS

Supplementary Materials for

Mindfulness-induced endogenous theta stimulation occasions self-transcendence and inhibits addictive behavior

Eric L. Garland *et al.*

Corresponding author: Eric L. Garland, eric.garland@socwk.utah.edu

Sci. Adv. **8**, eabo4455 (2022)
DOI: 10.1126/sciadv.abo4455

This PDF file includes:

Fig. S1

Figure S1. Theta power topomaps for patients in Mindfulness-Oriented Recovery Enhancement (MORE) and Supportive Group (SG) Psychotherapy at pre- and post-treatment.

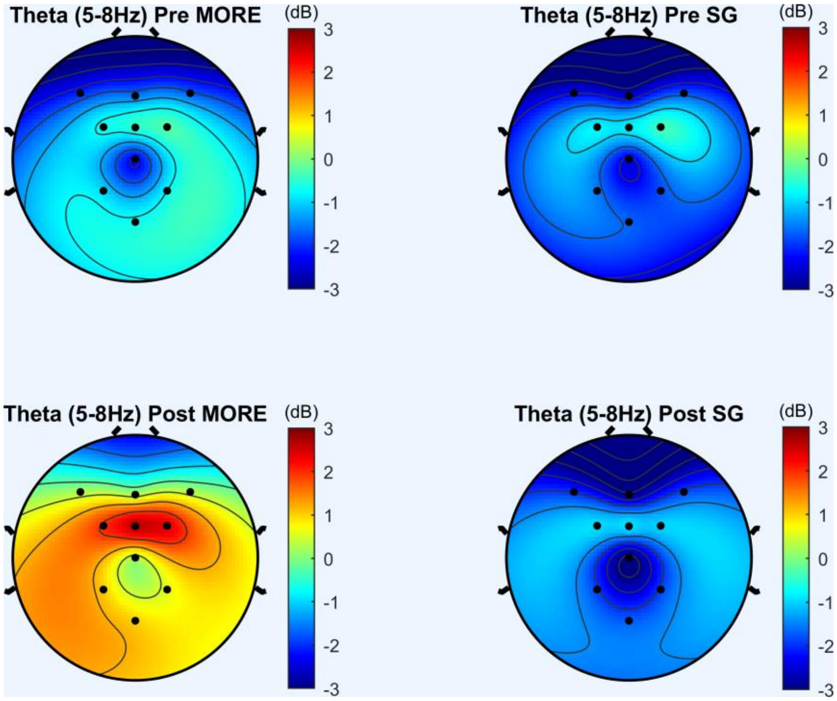

Supplement: Supplementary file 1 — Fig. S1 [file sciadv.abo4455_sm.pdf]
